# Decline of popliteal artery flow-mediated dilation with aging and possible involvement of asymmetric dimethylarginine in healthy men

**DOI:** 10.1007/s10396-019-00946-2

**Published:** 2019-04-23

**Authors:** Akihiro Nakamura, Shoko Kajitani, Kenjiro Sato, Masanori Kanazawa, Masateru Kondo, Hideaki Endo, Eiji Nozaki

**Affiliations:** 1 Department of Cardiology, Iwate Prefectural Central Hospital, 1-4-1 Ueda, Morioka, 020-0066 Japan; 2 Department of Cardiovascular Medicine, Tohoku University Graduate School of Medicine, Sendai, Japan

**Keywords:** Popliteal artery, Brachial artery, Flow-mediated vasodilation, Asymmetric dimethylarginine, Nitric oxide

## Abstract

**Purpose:**

We examined the influences of age and gender on flow-mediated endothelial function and the involvement of the competitive inhibition of l-arginine in endothelial function.

**Methods:**

We measured brachial and popliteal flow-mediated vasodilation (FMD) responses, nitrate/nitrite (NOx) concentrations, and plasma levels of asymmetric dimethylarginine (ADMA) in four healthy, nonsmoking groups: young men (mean 26 ± 2 years, *n* = 17), middle-aged men (mean 50 ± 3 years, *n* = 19), young women (mean 27 ± 2 years, *n* = 16), and middle-aged women (mean 51 ± 2 years, *n* = 18).

**Results:**

In young men, we found no significant differences between brachial and popliteal artery FMDs (10.6 ± 1.5 vs 8.7 ± 1.6%, *p* = 0.06). However, the popliteal artery FMD was significantly lower than the brachial artery FMD in middle-aged men (11.4 ± 1.5 vs 6.4 ± 1.0%, *p* < 0.001). In women, we found no significant differences between brachial and popliteal artery FMDs in young and middle-aged individuals (young, *p* = 0.17; middle-aged, *p* = 0.08). Popliteal artery FMD correlated with plasma NOx and ADMA levels as well as with the NOx/ADMA ratio in men but not in women (*r* = 0.485, − 0.544, and 0.672, respectively).

**Conclusion:**

We concluded that a decrease in flow-mediated endothelial function in arteries of the lower extremities was evident in healthy middle-aged men, but not in middle-aged women. The competitive inhibition of l-arginine may contribute to this decrease in men.

## Introduction

Atherosclerotic lesions are generally recognized as having a nonuniform distribution in the vasculature [1]. With regard to the extremities, it is a well-known fact that atherosclerotic lesions develop first in the arteries of the lower extremities before progressing to those of the upper extremities. This difference in the upper and lower extremities of humans is also supported by evidence of a higher incidence of atherosclerosis in the lower extremities, known as peripheral arterial disease (PAD) [2, 3].

Endothelium-dependent vasodilation plays an important role in the pathogenesis of many atherosclerotic diseases including PAD [4, 5]. Measurement of flow-mediated vasodilation (FMD) in the brachial artery by high-resolution ultrasound has been widely used as a noninvasive method for assessing endothelial function [5, 6]. The decrease in brachial artery FMD is used as a surrogate marker of cardiovascular function or a predictive marker of future cardiovascular events and has been observed in patients with cardiovascular disease [7] and hyperlipidemia [8]. Aging is a well-known risk factor for cardiovascular disease, and a consequence of aging is impaired vascular endothelial function [9, 10]. Although age-related declines in the vasodilator responsiveness of the brachial artery have been reported in healthy men without risk factors [11–13], the influence of aging on FMD responses in the lower extremities in healthy subjects has not been well studied. Gender differences have also been associated with the development of atherosclerosis, and brachial artery FMD in young women is greater than that in young and elderly men [11–15]. However, previous studies that examined the effect of gender differences on FMD responses have been limited to the brachial artery, and this effect has not been fully elucidated in the lower extremity arteries, especially in healthy subjects.

Endothelial dysfunction can also be assessed noninvasively by the measurement of circulating markers such as asymmetric dimethylarginine (ADMA), which is an endogenous and competitive inhibitor of nitric oxide (NO) synthase and acts as a regulator of the l-arginine/NO pathway [16, 17]. It has been shown that FMD is related to endothelium-derived NO bioavailability [18]. Although an inverse association between plasma ADMA levels and brachial artery FMD has been demonstrated not only in patients with atherosclerotic risk factors [19, 20] but also in healthy subjects [21, 22], the association between this marker and lower extremity artery FMD has not been well studied in healthy subjects.

We hypothesized that lower but not upper extremity artery FMD would be decreased in middle-aged subjects compared with young adults, and that plasma ADMA levels would be inversely associated with lower extremity artery FMD in healthy men and women. To test these hypotheses, using the brachial and popliteal arteries, we examined whether FMD responses in the upper and lower extremity arteries were affected by aging and gender, and whether an association between plasma ADMA levels and upper or lower extremity artery FMD existed in healthy men and women.

## Materials and methods

### Study subjects

Thirty-six healthy men (17 young and 19 middle-aged adults) and 34 healthy women (16 young and 18 middle-aged adults) volunteered to participate in this study. All subjects were nonobese (body mass index < 25.0 kg/m^2^), had normotensive resting blood pressure ≤ 140/90 mmHg, and had a negative smoking history. All subjects were normally active, were not taking any prescription medications, had clinically normal blood chemistry data [total cholesterol (TC) ≤ 240 mg/dl, low-density lipoprotein cholesterol (LDL-C) < 140 mg/dl, high-density lipoprotein cholesterol (HDL-C) ≥ 40 mg/dl, and hemoglobin A1c (HbA1c) < 5.7%], and had a resting supine ankle-brachial index (ABI) from 0.90 to 1.30. The subjects were free of obvious chronic diseases, as determined by a medical questionnaire and a physical examination including resting electrocardiogram. No subjects were taking any supplements with significant hemodynamic effects.

### Study protocol

We measured FMD for the assessment of endothelium-dependent vascular function in both the brachial and the popliteal arteries, and measured plasma ADMA levels for the assessment of endothelial dysfunction in all subjects. The subjects were instructed to fast overnight and were prohibited from food or exercise and from consuming caffeine, vitamins, or alcohol within 12 h before the start of the study. The study began at 9:00 a.m. The subjects were kept in a supine or seated position in a quiet, air-conditioned room with a stable temperature of 23 ± 1 °C during the study. At the beginning of the study, blood pressure and pulse rate were measured and blood sampling was performed. One hour after blood sampling, brachial artery FMD was measured. After the subject had maintained a seated position in the room for 2 h, popliteal artery FMD was measured. Both brachial and popliteal FMD were measured by one trained ultrasonographer blinded to the details of the study.

### Measurement of brachial and popliteal artery FMD

Brachial artery FMD [6] was measured with a semi-automated edge-detection system (UNEXEF18G; UNEX, Nagoya, Japan) according to the guidelines [23]. Briefly, the right brachial artery was scanned by high-resolution ultrasound with a 10-MHz linear array transducer for longitudinal and transverse images, while the subject was in a supine position after resting for ≥ 15 min. A sphygmomanometric cuff attached to the UNEXEF18G was positioned around the right forearm, and images of the artery were obtained on the proximal portion of the antecubital fossa. After recording of baseline images, the cuff was inflated to at least 50 mmHg above systolic blood pressure for 5 min and then deflated. The postdeflation arterial images were obtained in a similar manner as those for reactive hyperemia, and artery diameters were measured for 2 min with R-wave-synchronized automated edge-detection software. Brachial artery FMD was calculated as the maximal postdeflation diameter relative to the average baseline diameter.

Popliteal artery FMD was measured in a similar fashion to brachial FMD measurement using the UNEXEF18G [24]. After 2 h of rest following brachial FMD measurement, the subject was placed in a prone position. A sphygmomanometric cuff was positioned around the right calf, and the images of the artery at baseline were obtained on the proximal portion of the popliteal fossa. After reactive hyperemia produced by inflation of the cuff on the calf to at least 50 mmHg above systolic blood pressure of the leg for 5 min, continuous measurement of artery diameter was performed, and popliteal artery FMD was calculated with the same formula as that for brachial artery FMD: (maximal postdeflation diameter − baseline diameter)/(baseline diameter) × 100.

After 15 min of rest from brachial or popliteal FMD measurement, a resting scan was recorded and a sublingual nitroglycerin spray (0.3 mg; Toa Eiyo, Tokyo, Japan) was administered. The last scan was performed 3 min later. The change in diameter of the brachial or popliteal artery was expressed as percent dilation by nitroglycerin (NTG) (NMD: NTG-mediated vasodilation). To assess the reproducibility of brachial and popliteal FMDs with 80% power and α-type error of 5% in statistical analysis, a total of 60 measurements of brachial and popliteal artery diameter (each three times in 10 subjects) were intra-individually conducted. The coefficient of variation for repeated measurements of brachial and popliteal artery diameter at flow-mediated dilation was 1.38 ± 0.03 and 1.43 ± 0.02%, respectively, with this equipment.

### Blood sampling

Blood samples were obtained from the left antecubital vein with a 23-gauge polyethylene catheter, collected into heparinized tubes as plasma or into collection tubes containing serum separator gel as serum and immediately centrifuged to obtain the supernatant on the morning of the study (9:00 a.m.), 2 h before the measurement of brachial artery FMD. The plasma levels of NO metabolites (NOx: nitrite + nitrate) were measured at a contract laboratory (SRL, Tokyo, Japan), according to the Griess method. The samples were deproteinated and separated into nitrates and nitrites. After all the nitrates were reduced to nitrites, the samples were reacted with (*R*)-1-(1-napththyl)ethylamine, and the product was determined based on the absorbance at 540 nm [25]. The plasma levels of ADMA were also measured at SRL by high-performance liquid chromatography (HPLC): Hitachi L-7480 system equipped with a fluorescence detector for excitation at 348 nm and emission at 450 nm with an octadecyl-silica column using orthophthaldialdehyde for fluorescent determination [26]. The plasma levels of lipid parameters [TC, triglycerides (TG), LDL-C, HDL-C] and glucose parameters [fasting plasma glucose, HbA1c] were measured by standard methods (TC and TG by an enzymatic method; LDL-C and HDL-C by a direct method; HbA1c by HPLC) in our hospital laboratory.

### Statistical analysis

Values are expressed as means ± standard deviation for continuous variables and as numbers and percentages for categorical variables. Differences between two groups were assessed using Student’s unpaired *t *test or the Mann–Whitney *U* test for continuous variables and the chi-square test for categorical variables. Correlation between two parameters was determined by simple linear regression analysis. A two-sided *p* value ≤ 0.05 was considered to indicate statistical significance. All statistical analyses were performed with SPSS version 14.0 (SPSS, Chicago, IL, USA).

## Results

### Characteristics of study subjects

The characteristics of the subjects, 36 healthy male volunteers (17 young and 19 middle-aged men) and 34 healthy female volunteers (16 young and 18 middle-aged women), are summarized in Table 1. The mean ages of the young and middle-aged men were 26 ± 2 and 50 ± 3 years, respectively, while those of the women were 27 ± 2 and 51 ± 2 years, respectively. No subjects had a BMI ≥ 25 kg/m^2^ or a history of smoking. C-reactive protein was not significantly different among the four groups.

**Table 1:** Subject characteristics

Variable	Young men (*n* = 17)	Middle-aged men (*n* = 19)	*p* value	Young women (*n* = 16)	Middle-aged women (*n* = 18)	*p* value
Age, years	26 ± 2	50 ± 3	< 0.001	27 ± 2	51 ± 2	< 0.001
Height, cm	173 ± 5	172 ± 6	0.61	157 ± 7	157 ± 3	0.80
Weight, kg	64 ± 6	64 ± 6	0.88	48 ± 6	54 ± 8	0.16
BMI, kg/m^2^	22 ± 2	21 ± 1	0.55	19 ± 1	22 ± 3	0.058
Resting SBP, mmHg	120 ± 7	126 ± 12	0.20	111 ± 7	119 ± 13	0.15
Resting DBP, mmHg	66 ± 7	85 ± 12	0.003	65 ± 9	78 ± 7	0.006
Current or former smokers (*n*) (%)	0 (0)	0 (0)	1.00	0 (0)	0 (0)	1.00
Postmenopausal (*n*) (%)	–	–	–	0 (0)	10 (56)	< 0.001
Oral contraceptive use (*n*) (%)	–	–	–	0 (0)	0 (0)	1.00
CRP, mg/dl	0.12 ± 0.11	0.16 ± 0.12	0.33	0.11 ± 0.10	0.13 ± 0.10	0.53
Lipid markers						
Total cholesterol, mg/dl	197 ± 32	192 ± 34	0.88	187 ± 41	218 ± 21	0.032
Triglycerides, mg/dl	89 ± 36	64 ± 34	0.25	54 ± 26	75 ± 36	0.23
LDL-cholesterol, mg/dl	110 ± 26	109 ± 39	0.93	91 ± 13	113 ± 25	0.021
HDL-cholesterol, mg/dl	67 ± 13	70 ± 19	0.58	85 ± 32	90 ± 18	0.72
Glucose markers						
FPG, mg/dl	94 ± 10	104 ± 12	0.086	96 ± 7	96 ± 10	0.90
HbA1c, %	5.3 ± 0.3	5.5 ± 0.5	0.19	5.3 ± 0.3	5.6 ± 0.2	0.022
Endothelial biomarkers						
NOx, µmol/l	40.3 ± 17.2	29.7 ± 14.5	0.098	49.1 ± 11.4	40.8 ± 14.5	0.076
ADMA, µmol/l	0.34 ± 0.09	0.39 ± 0.06	0.052	0.34 ± 0.06	0.35 ± 0.04	0.496
NOx/ADMA	125.3 ± 49.2	81.5 ± 47.2	0.010	152.2 ± 49.0	120.3 ± 49.5	0.069

Data are presented as means ± SD for continuous variables and as percentages for categorical variables

*BMI* body mass index, *SBP* systolic blood pressure, *DBP* diastolic blood pressure, *CRP* C-reactive protein, *LDL* low-density lipoprotein, *HDL* high-density lipoprotein, *FPG* fasting plasma glucose, *HbA1c* hemoglobin A1c, *NTG* nitroglycerin, *NOx* nitrate + nitrite, *ADMA* asymmetric dimethylarginine

### Concentration of plasma NOx and ADMA

Plasma NOx levels were lower in middle-aged men and women than in young men and women, although these differences were not statistically significant (men 29.7 ± 14.5 vs 40.3 ± 17.2 µmol/L, *p* = 0.098; women 40.8 ± 14.5 vs 49.1 ± 11.4 µmol/L, *p* = 0.076). Plasma ADMA levels were higher in middle-aged men than in young men, although this difference was not statistically significant (0.39 ± 0.06 vs 0.34 ± 0.09 µmol/L, *p* = 0.052). Plasma ADMA levels were similar in young and middle-aged women (0.34 ± 0.06 vs 0.35 ± 0.04 µmol/L, *p* = 0.496). The ratio of plasma NOx levels to ADMA levels was significantly lower in middle-aged men than in young men (81.5 ± 47.2 vs 125.3 ± 49.2, *p* = 0.010), but was not significantly different between young and middle-aged women (152.2 ± 49.0 vs 120.3 ± 49.5, *p* = 0.069) (Table 1).

### FMD of brachial and popliteal arteries in men and women

Three groups (young men, middle-aged men, and middle-aged women) had significantly greater baseline vessel sizes in the popliteal artery than in the brachial artery (young men 5.00 ± 0.48 vs 3.88 ± 0.33 mm, *p* < 0.001; middle-aged men 5.80 ± 0.10 vs 3.97 ± 0.41 mm, *p* < 0.001; middle-aged women 4.06 ± 0.64 vs 3.31 ± 0.38 mm, *p* = 0.005). In young women, baseline vessel size was similar in the brachial and popliteal arteries (3.43 ± 0.53 vs 3.73 ± 0.61 mm; *p* = 0.20) (Table 2). The baseline vessel size of the popliteal arteries was significantly greater in middle-aged men than in young men (5.80 ± 0.10 vs 5.00 ± 0.48 mm, *p* = 0.016). There was no significant difference in the basal vessel size of the popliteal arteries between young and middle-aged women (3.73 ± 0.61 vs 4.06 ± 0.64 mm, *p* = 0.304).

**Table 2:** Brachial vs popliteal artery measurements in men and women

Measurement	Young men (*n* = 17)	Middle-aged men (*n* = 19)	*p* value	Young women (*n* = 16)	Middle-aged women (*n* = 18)	*p* value
Baseline diameter, mm						
Brachial artery	3.88 ± 0.33	3.97 ± 0.41	0.27	3.43 ± 0.53	3.31 ± 0.38	0.34
Popliteal artery	5.00 ± 0.48^§^	5.80 ± 0.10^§^	0.016	3.73 ± 0.61	4.06 ± 0.64^†^	0.30
FMD, %						
Brachial artery	10.6 ± 1.5	11.4 ± 1.5	0.12	12.9 ± 1.3	11.9 ± 2.4	0.15
Popliteal artery	8.7 ± 1.6	6.4 ± 1.0^§^	< 0.001	11.5 ± 2.0	10.5 ± 2.0	0.18
NMD, %						
Brachial artery	17.6 ± 2.3	18.1 ± 2.7	0.38	19.7 ± 2.5	19.2 ± 3.4	0.71
Popliteal artery	16.4 ± 2.9	15.9 ± 3.1	0.45	18.6 ± 2.3	17.9 ± 3.1	0.52
FMD/NMD ratio, %						
Brachial artery	67.7 ± 9.9	63.8 ± 7.4	0.18	68.3 ± 7.6	62.5 ± 7.2	0.53
Popliteal artery	56.3 ± 5.7^§^	42.8 ± 4.1^§^	< 0.001	59.8 ± 5.9^†^	56.1 ± 6.6^†^	0.10

Data are presented as means ± SD

*FMD* flow-mediated vasodilation, *NMD* nitroglycerin-mediated vasodilation

^†^*p* < 0.01 and ^§^*p* < 0.001 comparing brachial and popliteal artery, respectively

Table 2 shows brachial and popliteal artery FMD and NMD in the four groups. In young men, no significant difference was observed between brachial and popliteal artery FMD (10.6 ± 1.5 vs 8.7 ± 1.6%, *p* = 0.06). In middle-aged men, there were significantly smaller FMD values in the popliteal artery than those in the brachial artery (6.4 ± 1.0 vs 11.4 ± 1.5%, *p* < 0.001). No significant differences were observed between brachial and popliteal artery FMD in either young or middle-aged women (young women 12.9 ± 1.3 vs 11.5 ± 2.0%, *p* = 0.17; middle-aged women 11.9 ± 2.4 vs 10.5 ± 2.0%, *p* = 0.08). The values of NMD in the brachial and popliteal arteries were not different among the four groups (young men 17.6 ± 2.3 vs 16.4 ± 2.9%, *p* = 0.57; middle-aged men 18.1 ± 2.7 vs 15.9 ± 3.1%, *p* = 0.28; young women 19.7 ± 2.5 vs 18.6 ± 2.3%, *p* = 0.41; middle-aged women 19.2 ± 3.4 vs 17.9 ± 3.1%, *p* = 0.33). FMD/NMD ratios were significantly smaller in the popliteal arteries than in the brachial arteries in the four groups. These ratios in the popliteal arteries were significantly smaller in middle-aged men than in young men (42.8 ± 4.1 vs 56.3 ± 5.7%, *p* < 0.001); however, there were no significant differences between young and middle-aged women.

### Correlation between plasma NOx and ADMA levels and FMD

**Fig. 1:**
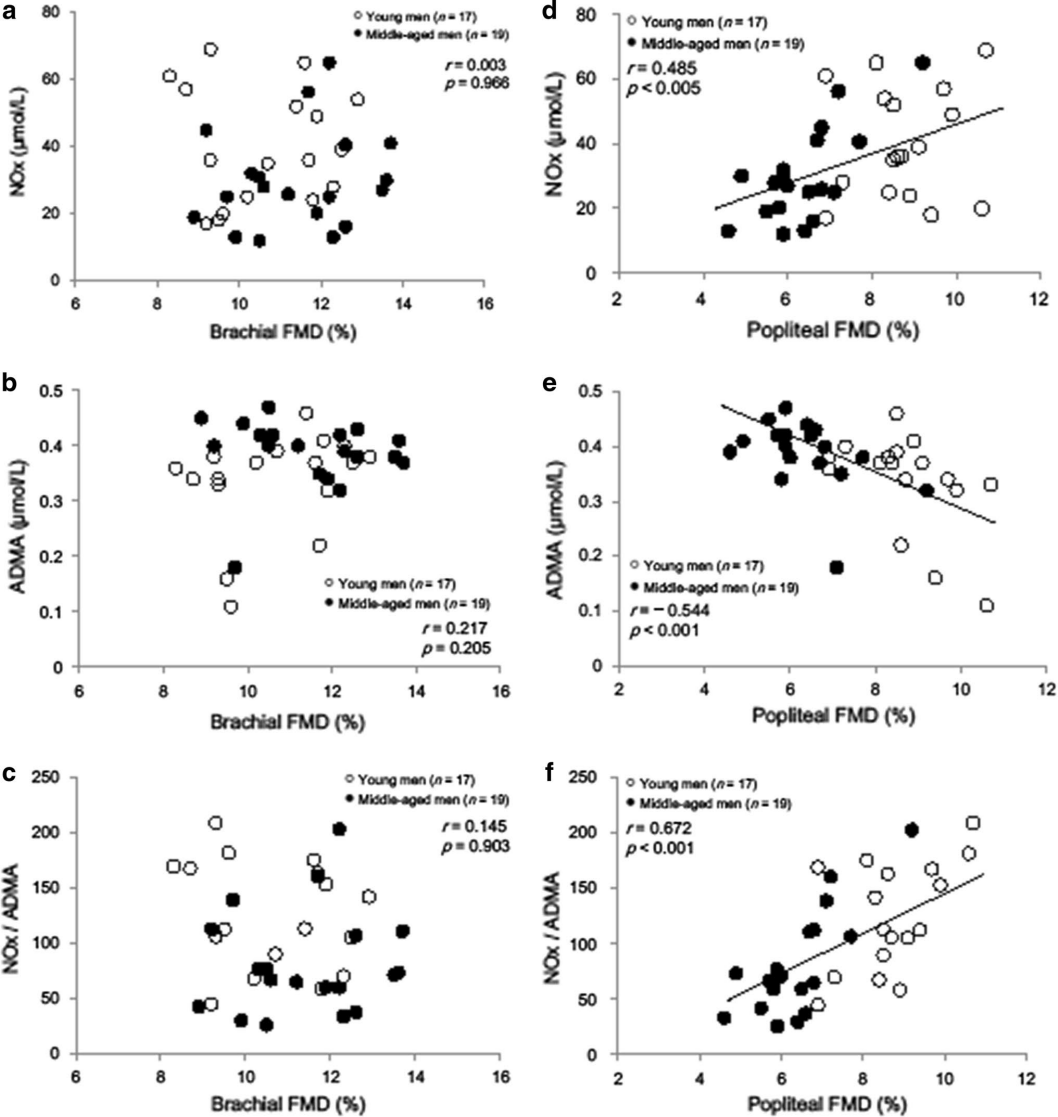
Correlations between FMD and plasma NOx, ADMA, and NOx/ADMA ratio in men. Left panels showing the relationship between brachial FMD and NOx (**a**), ADMA (**b**), and NOx/ADMA ratio (**c**); right panels showing the relationship between popliteal FMD and NOx (**d**), ADMA (**e**), and NOx/ADMA ratio (**f**). *FMD* flow-mediated dilation, *NOx* nitrate + nitrite, *ADMA* asymmetric dimethylarginine

Figure 1 illustrates the correlation between plasma NOx and ADMA levels and brachial and popliteal artery FMD in men. The plasma levels of NOx and ADMA showed a good correlation with popliteal artery FMD (Fig. 1d, e), but not with brachial artery FMD (Fig. 1a, b), in men. The NOx/ADMA ratio correlated especially well with popliteal artery FMD (Fig. 1f), but not with brachial artery FMD (Fig. 1c). In women, both brachial and popliteal FMD did not correlate with the plasma levels of NOx and ADMA (popliteal FMD and NOx: *r* = 0.14, *p* = 0.89; popliteal FMD and ADMA: *r* = 0.036, *p* = 0.84).

## Discussion

FMD measurements in previous studies were performed on either the upper or the lower extremity arteries. Unique to this study was the fact that we could investigate FMD in both the upper and lower extremity arteries of healthy subjects on the same day. This study also investigated the effects of plasma NOx and ADMA levels on upper and lower extremity artery FMD with respect to age and gender. The main findings of this study were that popliteal artery FMD did not significantly differ from brachial artery FMD in healthy young men or in healthy young and middle-aged women, but was significantly smaller than brachial artery FMD in healthy middle-aged men; and that popliteal artery FMD correlated well with plasma NOx and ADMA levels and with the NOx/ADMA ratio in men, but not in women. These findings suggest that the decline in vascular endothelial function associated with aging differs between leg and arm conduit arteries in healthy men, but not in women younger than the early 50s.

Aging is recognized as an independent risk factor for the development of atherosclerosis [27]. Age-related hemodynamic and anatomic changes in the cardiovascular system are known to be associated with functionally impaired vascular responses, particularly endothelium-dependent vasodilation [11, 28]. Previous studies have demonstrated that the effect of aging on FMD response in the upper extremities differs between healthy men and women. Corretti et al. [28] showed that endothelium-dependent vasoactivity differentiated older (> 40 years of age) men from younger (< 40 years of age) normal men based on the FMD response in the brachial artery, and that this response did not differentiate normal older premenopausal (> 40 years of age) women from younger (< 40 years of age) normal women. Celermajer et al. [11] showed that an age-related decline in brachial artery FMD occurred in women starting from 53 years of age. Our finding showing no difference in brachial artery FMD between young and middle-aged healthy women supports the stability of brachial artery FMD responses in healthy women until the early 50s. When young and middle-aged healthy men were compared, there was no significant difference in brachial artery FMD in our study. This finding was compatible with that of other studies showing similar brachial FMD responses in Swedish men at 35 and 55 years of age [29]. Although the starting age of endothelial-dependent vasodilation decline in men was reported from 41 years of age [11], our study suggests that it may be later than that age.

The nature of the vasculature is nonuniform, and arteries in the lower extremities are more sensitive to the development of atherosclerosis than those in the upper extremities. Arterial diseases typified by PAD are more common in lower extremity arteries than in upper extremity arteries, and they are highly correlated with the progression of coronary artery diseases [30]. Although the upper extremities are well-established clinical research models for noninvasive assessment of endothelial function, they are of limited consequence to systemic cardiovascular control because of their small muscle volume and blood flow requirements. Measurement of FMD in the lower extremity arteries would thus provide meaningful information for better understanding of the effects of aging on endothelial function. Although several clinical studies have revealed attenuated vascular endothelial responses in the lower extremities of subjects with cardiovascular diseases [31], there is a paucity of FMD studies regarding age-related declines in vascular function in lower extremity arteries [32].

Our study demonstrated that endothelial flow-dependent dysfunction with aging was greater in lower extremity arteries than in upper extremity arteries in healthy men. Nishiyama et al. [32] reported that the absolute values of FMD did not differ between the brachial and popliteal arteries in young healthy subjects (mean age 26 ± 2 years), whereas those in the popliteal artery were significantly attenuated compared with the brachial arteries in older healthy subjects (mean age 72 ± 1 years). We examined popliteal artery FMD in middle-aged men (mean age 50 ± 3 years) compared with that in young men (mean age 26 ± 2 years). The age of healthy men showing a decline of leg artery endothelial function was lower than that of subjects demonstrated before by Nishiyama et al. [32]. In women, there were no significant differences in FMD between young and middle-aged healthy subjects in the brachial and popliteal arteries. These findings, which are compatible with those of previous studies [24], suggest that FMD is preserved until the early 50s not only in the brachial arteries but also in the popliteal arteries. However, the exact reason why the decrease in popliteal artery FMD was not observed in women is still unclear. The effect of sex hormonal factors may best explain the gender difference in age-related decline in endothelial flow-dependent function. Estrogens have been shown to protect premenopausal women from the decrease in endothelial function via an increase in blood flow and subsequently shear stress [33]. They have also been reported to be associated with a lower production of reactive oxygen species (ROS), which were found to contribute to arterial stiffening in estrogen-deficient postmenopausal women [34]. In this study, all middle-aged women were in their early 50s (mean 51 ± 2 years), and about half of them were postmenopausal. Although it is unknown how long the protective effects of estrogens against endothelial injury last after menopause, it seems that the women at the age around the time of menopause are protected from the development of endothelial flow-mediated dysfunction with aging.

In this study, there was a difference in baseline diameters of popliteal arteries between young and middle-aged men. Baseline arterial diameters, which are part of the calculation of percentage FMD, have been reported to influence FMD responses by impacting shear stress on the arterial wall [35]. It is unclear whether FMD responses might be influenced by the structural differences, but the decline in popliteal artery FMD in men could be caused by endothelial flow-dependent dysfunction because of no significant differences in popliteal artery NTG-mediated vasodilation between young and middle-aged men.

This study demonstrated that aging is related to a decrease in endothelial function in healthy men, partially manifesting as impaired popliteal FMD. However, as the underlying mechanism remains unclear, we also measured FMD together with plasma NOx and ADMA levels. NO, which is produced within the endothelium by the enzyme NO synthase (NOS) with l-arginine as a substrate, regulates many antiatherogenic effects such as vasodilation and modulation of vascular smooth muscle cells [36]. It also modulates many processes involved in aging. Sverdlov et al. [9] recently reported that aging was independently associated with impaired NO generation and its effects. It has also been demonstrated that plasma ADMA levels have a good correlation with age and reflect a vascular degenerative process associated with aging [37]. Our study showed that plasma NOx or ADMA levels were not significantly different by age or gender (see Table 1). However, when comparing young and middle-aged subjects, the NOx/ADMA ratio was significantly smaller in middle-aged men or tended to be smaller in middle-aged women. As previously reported [38], the ratio may be more sensitive to endothelial dysfunction. Although the relationship between plasma NO or ADMA levels and aging has been studied, little information is available regarding the correlation between FMD responses and plasma levels. Casey et al. [39] reported that systemic plasma NOx levels reflected brachial artery FMD responses, and Lauer et al. [40] reported that older subjects had impaired responses for brachial artery FMD and lower levels of plasma NOx compared with younger subjects. It has also been demonstrated that plasma ADMA levels have an inverse association with brachial artery FMD in healthy subjects [21, 41, 42]. Contrary to these reports, Gates et al. [43] reported that the expression of endothelial cell ADMA protein was not higher in older adults than in younger adults, and they suggested that competitive inhibition of l-arginine binding sites on endothelial NOS by ADMA was not an important mechanism contributing to age-related loss of brachial artery FMD responses in healthy subjects. Consistent with their report, we also observed that brachial artery FMD was not correlated with plasma NOx or ADMA levels in healthy men and women. Of particular interest was the observation that good correlations were found between these plasma levels and popliteal artery FMD in men. This finding suggests that impaired endothelial function in the leg conduit artery with aging may be at least partially explained by NO bioavailability or ADMA levels in men, but not in women. In women, endothelial flow-dependent function may be modulated by other additional factors without plasma NOx and ADMA. Mendelsohn [44] reported that estrogens preserved endothelial-dependent vasodilatory function by increasing NO release and by protecting NO from inactivation by ROS. Estrogens have also been reported to inhibit the synthesis of endothelin-1, which is a vasoconstrictor that opposes the effects on NO [45]. Moreau et al. [46] reported that plasma endothelin-1 concentrations were lower in premenopausal compared with postmenopausal women. Thus, the favorable effect of sex hormones may be associated in part with preserved FMD responses in women, and this may be the reason why both brachial and popliteal artery FMD of women did not correlate with plasma NOx and ADMA.

There were several limitations to the present study. First, a relatively small number of subjects were evaluated. Second, we used first-generation equipment to measure the FMD responses, and we could not assess shear stress after deflation of the cuff. Since shear stress is closely related to FMD responses, the normalization of FMD with shear stress may contribute to better understanding of the differences in brachial and popliteal endothelial function with aging. Finally, we examined the association between brachial and popliteal artery FMD responses and plasma ADMA levels that were measured as systemic circulating protein. However, systemic plasma ADMA levels may not reflect local ADMA levels in the tissues, where ADMA may have more potent and direct regulatory roles. We cannot estimate how ADMA expressed in leg or arm arteries is associated with each endothelial-dependent dilation function.

## Conclusion

We demonstrated that popliteal artery FMD was significantly lower than brachial artery FMD in healthy middle-aged men, but not in young men or women. We also observed that popliteal artery FMD showed a negative correlation with plasma ADMA levels and a positive correlation with plasma NOx levels as well as with the ratio of plasma NOx and ADMA levels in healthy men, but not in women. These findings suggest that competitive inhibition of l-arginine by ADMA may lead to impaired popliteal artery endothelium-dependent dilation in healthy men until the early 50s.
